# The Penn Mutual Life Insurance Company, Philadelphia

**Published:** 1906-05

**Authors:** 


### CORRESPONDENCE.

### THE PENN MUTUAL LIFE INSURANCE COMPANY, PHILADELPHIA.

### LETTER OF DR. REX.

DEAR DOCTOR: We have received from certain State and county medical societies copies of resolutions fixing the fee for life insurance examinations, conflicting in some particulars with the rule of this company.

Our compensation for examinations is graded *according to the amount of work required and not according to the amount of the policy*. The line is distinctly and sharply drawn by us on this point. Our reason for establishing the rule of no urine test under certain conditions and maintaining it is, that we long ago found that the premium collected on $1,000 cases, a great many payable quarterly and on the cheapest form, was practically exhausted by initial expense. We, therefore, concluded to drop the urine test in $1,000 cases under age fifty, our mortality experience indicating that this could be safely done. The unit of compensation for this test with us has always been two dollars, and where not needed it represents a saving of this amount. Our rule is universal in the United States, is and has been acceptable to some thirty thousand examiners since its inauguration fifteen years ago. It would be an injustice to discriminate between our examiners, paying a larger fee in one section than in another. It would be unfair to compel our solicitors to pay this excess fee. We feel sure that our position is the correct one, and trust that you will see it in this light. Where the question has been raised, the justice of our contention has been admitted by prominent officers of both State and county medical societies. We are not antagonistic to these organizations, but we do not think that they should intentionally encourage their members as a body to disregard an agreement which every regularly appointed examiner for this company has signed, to the effect that they will make examinations strictly in accordance with the instructions printed on the medical examiners' certificate. Our examiners received individually separate letters of instruction covering the schedule of fees paid by this company. No change has been made by us nor did we expect them to disregard their agreement. Some medical societies discriminate against us by excluding fraternal associations from their resolutions. We have never been nor are we now in combination with other companies to fix fees. Our examiners necessarily represent a minority in each county medical society. Should the question ever arise in your society, would you support an amendment to the resolutions to the effect that the examiners for the Penn Mutual have no just cause for complaint, and that they be permitted to continue on the old basis without prejudicing their positions with the county society?

Since March 2, 1906, we have learned that in North Carolina, Bertie, Craven, Gaston, Johnston, Rowan, Transylvania, Vance, Wayne and Yadkin counties have *modified* their resolutions to the effect that *when urine analysis is not required, the fee shall be* ($3.00) *three dollars.*

Alexander, Ashe, Bertie, Brunswick, Burke, Cabarrus, Catawba, Chowan, Edgecombe, Henderson, Iredell, Jackson, McDowell, Madison, Mecklenburg, Mitchell, Pasquotank, Perquimans, Randolph, Surrey, Union and Warren counties have not taken definite action, but our reports indicate that our examiners agree with us and a modified resolution will be favored.

In Cleveland county our examiners refused to abide by the unmodified resolution of the society.

About 98 per cent. of the examiners in North Carolina from whom we have heard are in entire accord with us.

If you can do anything to put this matter before your county society in the proper light, we will greatly appreciate it. We will thank you for an expression of your views on these points and the courtesy of an early reply.

Yours truly, O. P. REX,

No. 479. Medical Director.

REPLY.

MACON, GA., April 2, 1906.

*Dr. O. P. Rex, Medical Director, Penn Mutual Life Insurance Company, Philadelphia, Pa.*

MY DEAR DOCTOR: Replying to your circular letter No. 479, in regard to life fees, I will refer to the status of the question in this city only, as I am quite familiar with it. Personally I have never knowingly made an examination for less than five dollars, and do not intend to.

In 1865 the Macon Medical Association passed a resolution to charge five dollars for life examinations. If any member has made one for less since that date, he lied, and this I question. As your company and others have notified their examiners of the reduction of fees, they have promptly received their resignation, as you may recall. No man in this community in good professional standing has examined on the cut-rate basis, and I hope they never will.

Of course in every community of fifty thousand people you will find men who, for various and obvious reasons not necessary to enumerate, will make examinations for any fees, even twenty-five cents, as has been done here. Where every man's personal and professional status is known, you can easily surmise what effect it may have on the standing of the company to employ men of this type. Your position is such that you know what qualifications a doctor has to have to make a first-class examination and what amount of time it takes.

In my opinion, a properly-made life examination for five dollars is the smallest fee we receive in this city for the service rendered.

You claim that it would be unjust to your examiners to pay different fees in different places. Other companies claim that they pay five dollars here and other fees elsewhere.

Our office fee is $2.00, consultation $15.00, obstetrics $30.00. Should we cut them to 50 cents, $2.50 and $5.00 respectively because there are places where these services are rendered at the above rates?

No physician here is absolutely dependent on life insurance examinations, but we are all dependent on our professional reputation. I examine for six or eight companies, and it is of course a considerable item in my income. At the same time, I would resign from every one before I would recommend an applicant for insurance whose urine I had not examined. In other words, I would not sign an application when I had not made a complete examination of the patient, no matter what the requirements of the company might be. I have too much respect for my reputation to take any such risks.

Whether it is just to your policyholders present and future to accept an applicant for a thousand dollar policy who might be rejected under your requirements for $1,100 policy is, of course, a question in which I have no interest.

There are many cases where a doctor has lost more in reputation from one examination than he could make from life insurance in his entire professional career. It goes without argument that you have the right to fix your fee, and that the profession has the equal right to accept the same or not.

The better element of the profession here think that $5.00 should be minimum fee for a proper examination, and are too honest to make any other kind. Elsewhere this fee might be too large or too small, dependent on local conditions.

What effect this reduction may have had on your statistics I, of course, do not know, but I do know that in my own restricted field of observation I have seen over $75,000 paid out in death claims on small or moderate sized policies, not one of which could by any chance have passed any one of the corps of examiners that you had in this city fifteen years ago.

In pursuance of your request, I shall take pleasure in bringing this matter before such societies as I can as opportunity may offer.

Very truly yours,

H. MCHATTON,

Ex-President Medical Association of Georgia,

President Macon Medical Association.

RESOLUTIONS.

At regular meeting of Macon Medical Society of Bibb County, the following resolutions were unanimously adopted:

*Resolved, first,* That the secretary be directed to write Dr. O. P. Rex that his letter to Dr. McHatton has been read to this Society, and the Society, by unanimous vote, consider it the most absurd request ever made by a reputable medical man to a body of medical gentlemen, and it has made the members of this Society more convinced that they are right in the stand they have taken since 1865 concerning examination fees.

*Second,* That a copy of these resolutions, with Dr. O. P. Rex's letter and reply thereto, be published in *Georgia Practician* and ATLANTA JOURNAL-RECORD OF MEDICINE.

---

### COMPLICATIONS OF OVARIAN TUMORS WITH SPECIAL REFERENCE TO MALIGNANCY.

Norris summarizes his paper in the following propositions: One in every four to six ovarian tumors is malignant, and this proportion warrants that all ovarian tumors be regarded as malignant until the contrary is proven. The operative mortality in such malignant disease should not be more than 10 or 12 per cent. The cures at the end of five years will be few, carcinoma being the most frequent and dangerous of malignant diseases of the ovary. Every ovarian tumor should be operated upon as soon as discovered, if possible. "Borline" should be operated upon; there may be clinical evidences of malignancy and the tumor prove benign, or the reverse. Exploratory operations will often give comfort by removing the pressure made by ascitic fluid, and should always be performed in cases in which the diagnosis is in doubt. All specimens should be examined rigorously and extensively by the microscope. Parovarian are less likely to be malignant than ovarian cysts. Torsion is next in frequency to malignancy as a complication.—*American Journal of Obstetrics, January, 1906.*

